# Sir William Roberts on Digestion

**Published:** 1904-11

**Authors:** 


### SIR WILLIAM ROBERTS ON DIGESTION.

Sir William Roberts, of London, the great authority on digestion, says: "The digestive change undergone by fatty matter in the small intestines consists mainly in its reduction into a state of emulsion or division into infinitely minute particles. In addition to this purely physical change, a small portion undergoes a chemical change whereby the glycerine and fatty acids are dissociated. The main or principal change is undoubtedly an emulsifying process, and nearly all the fat taken up by the lacteals is simply in a state of emulsion."

This eminent authority is confirmed in the foregoing view by various experiments by which it has been ascertained that fat foods pass from the lacteals into the circulation by way of the thoracic duct in the form of an emulsion.

Emulsified cod liver oil as contained in Scott's Emulsion appears in a form so closely resembling the product of natural digestion—as it occurs within the body—that it may be well administered as an artificially digested fat food of the very highest type. In combination with the other ingredients mentioned—glycerine being an emollient of estimable value—Scott's Emulsion offers to the physician a valuable, exquisite and rare accession to his prescription list.

